# 

**Published:** 1888-06

**Authors:** 


CONTENTS

VOL. 35 No. 6

| | PAGE. |
|---|---|
| Mind Cure | 121 |
| The New World Language | 122 |
| Mind Cure, No. 2 | 126 |
| Foods and Beverages | 131 |
| A New Process for Preserving Meats and Liquids | 133 |
| Prof. Severance tells how he keeps from ever getting sick | 134 |
| Disease in Milk | 135 |
| The Traveler's Tree | 137 |
| Ice Houses | 138 |
| Theological Scarecrows | 138 |
| A Dream Fulfilled | 139 |
| Curiosities of Foods | 139 |
| Book Notices | 140 |
| Household | 141 |
| Miscellaneous | 143 |

INDEX TO ADVERTISEMENTS OF HALF A PAGE AND OVER.

| | PAGE. |
|---|---|
| Crystal Pepsin Tablets | I |
| Stevensville Mills | II |
| The Tea Cure | II |
| The American Oxygen Association | III |
| Hollow Suppositories | IV |
| Lactopeptine | V |
| Premiums of Books | VI. |
| Premiums of Silver Plate | VII |
| Bovinine | VIII |
| Volapuk | IX |
| Aletris Cordial | X |
| Celerina | X |
| The Welsbach Incandescent Gas Burner | XI |

